# A New View of the Waggle Dance: Making Scents to Recruit Fellow Foragers

**DOI:** 10.1371/journal.pbio.0050249

**Published:** 2007-08-21

**Authors:** Liza Gross

To the untrained observer, the collective feats of social insects appear downright mysterious. How could insects with decidedly less-complex neural circuits than humans evolve the highly sophisticated communication strategies needed to build 3- to 4-meter-tall mound nests (termites), haul vegetation back to the home colony to cultivate fungi for food (leafcutter ants), or launch mass predatory attacks on other social insects like bees or wasps (giant hornets)? Though the past quarter century has yielded critical insights into the molecular and physiological underpinnings of insect sociality, scientists have yet to elucidate key aspects of social behavior and its regulation.

The honeybee and its waggle dance emerged as a model of animal communication and social behavior in 1947, when Karl von Frisch discovered that waggling tells other bees where to find bountiful forage away from the hive. Biologists have been trying to figure out how wagglers convey this information and attract recruits in the darkness of the hive ever since. Sounds, comb vibrations, tactile cues (from touching antennae), and odors all play some role in this elaborate transfer of information, but none of these signals can fully explain how foragers are recruited.

Now in a new study, Corinna Thom et al. report yet another component of the waggling honeybee’s lexicon. Waggle-dancing bees, the researchers found, produce four distinctive odors that induce foraging activity in other honeybees. Previous studies have shown that odors picked up while foraging may help recruits pinpoint a rich food source, but this is the first evidence that wagglers emit olfactory compounds (called semiochemicals) that influence the behavior of other foragers. Based on evidence that honeybees (and other social insects) use pheromones to organize colony mates for hive tasks, the researchers reasoned that olfactory cues may also play an important role in coordinating foraging activity.

They tested this hypothesis by sampling the airspace around waggle dancers and nondancing bees with filters designed to extract volatile compounds from the air. Waggle dancers, they discovered, emitted four compounds that were absent from the airspace of nondancing bees. Compared with nondancing foragers and nonforaging bees, wagglers also had significantly higher amounts of these compounds, called cuticular hydrocarbons, on their abdomens. Cuticular hydrocarbons, produced beneath the insects’ outer covering (called the cuticle), act as recognition cues in social insects, signaling nest mates and castes, for example. Interestingly, the most energetic waggle dancers produced the most copious quantities of these compounds.

**Figure:**
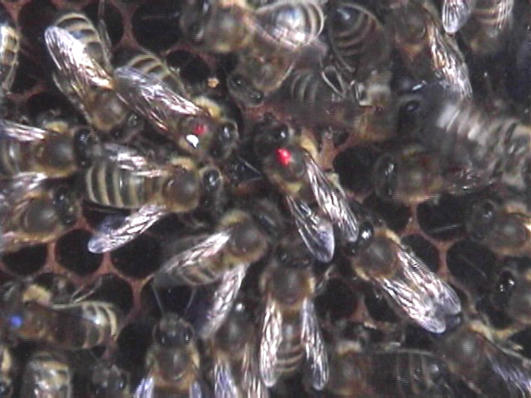
Waggle-dancing bees (center, with red mark) release scents that recruit other foragers.

To find out whether the isolated chemicals influenced behavior, the researchers injected one of two mixtures onto the honeybee “dance floor” and then counted the number of bees exiting the hive. The first mixture contained three of the isolated compounds (only three are commercially available) dissolved in a solvent (hexane). The second mixture contained only the solvent. (To control for any confounding differences between the treatment and solvent injections, they also ran trials that alternated between two hexane solvent injections.) Bees exposed to the treatment mixture of the three hydrocarbons left the hive in far greater numbers than bees exposed only to the hexane injections.

Altogether, these observations indicate that pheromones unique to waggle-dancing bees activate foraging behavior in the hive, revealing an unappreciated role for semiochemicals in waggle-dance communication. The researchers propose that synthesis of the compounds increases in dancing bees and that the waggle-dance scent may be released as dancing raises their body temperature.

Though waggle-dance scent compounds have been associated with specific behaviors in other insects—for example, *Vespula* wasps use them to establish and follow trails, and flies use them as sex attractants—the compounds had not been linked to specific honeybee behavior before. These new results suggest that the waggle scent serves as an attractant for honeybees, too. The scent of wagglers may serve to identify them as successful provisioners and attract the attention of recruits, who home in on the dancing foragers for details on the food site. It could then induce the hive to quickly organize a profitable foraging effort. The waggle scent may even convey trip-related details to recruits who are temporarily jostled away from the dancer. With evidence for a new role for olfactory cues in symbolic animal communication, researchers can explore just how the waggle scent elicits a behavioral response—and continue to decode the myriad communication mechanisms underlying the cooperative exploits of social insects.

